# Media suicide-reports, Internet use and the occurrence of suicides between 1987 and 2005 in Japan

**DOI:** 10.1186/1471-2458-7-321

**Published:** 2007-11-11

**Authors:** Akihito Hagihara, Kimio Tarumi, Takeru Abe

**Affiliations:** 1 Kyushu University Graduate School of Medicine, Department of Health Services, Management and Policy, Higashi-ku, Fukuoka 812-8582, Japan; 2 University of Environmental and Occupational Health, Post Graduate Guidance Section, 1-1 Iseigaoka, Kita-kyushu 807-8555, Japan

## Abstract

**Background:**

Previous investigations regarding the effects of suicide reports in the media on suicide incidence in Japan have been limited and inconclusive and, although Internet use has greatly increased, its influence on suicide is completely unknown. Thus, the relationship between newspaper articles about suicide, Internet use, and the incidence of suicide in Japan was examined.

**Methods:**

A linear model was fitted to time series data from January 1987 to March 2005 (218 months).

**Results:**

Consistent with previous findings, the number of newspaper articles about suicide was a predictor of suicide among both male and female subjects. Internet use was also a predictor of suicide among males, probably because males spent more time online than females.

**Conclusion:**

Because this is the first, preliminary study examining the association between Internet use and suicide, further research is required to verify the present findings.

## Background

Numerous studies into the effect of suicide reporting on the incidence of suicide have been conducted in the United States and Europe [1-4]. According to Stack [5], there were 293 investigations concerning suicide news reports and suicide as of 1999. Although the findings have been mixed, the overall trend indicates that the media does have an impact on suicide [6-10]. Therefore, it is widely believed that suicide reports in the Japanese media lead to an increased number of suicides. In April 1986, an 18-year-old female singer committed suicide in Japan. The singer was very popular among the younger generation, especially teenagers, and news of her suicide came as a great shock. However, the coverage of this event was considered by many to be excessive. This prompted the Japanese Suicide Prevention Association, who were aware of the danger of suicide reporting, to send letters to media companies requesting that they cease their coverage [11].

The existing information on the effects of suicide reporting on suicide in Japan are very limited and inconclusive. As of July 2005, only eight studies had been conducted, and four of these compared the incidence of juvenile suicide before and after the suicide of the aforementioned female singer on 8 April 1986 [5,11-17]. Yoshida *et al*. compared the observed and expected numbers of suicides in the under-20 age group after April 8, 1986. The expected number of suicides was calculated using a Poisson distribution and the observed number of suicides in the under-20 age group prior to 8 April 1986. Because the observed number of suicides was significantly higher than the expected number, the authors concluded that suicide reporting did have an effect on suicide incidence [11,17]. Kurusu reported the daily number of suicides after April 8, 1986 among four age groups (i.e., 10–14, 15–19, 20–24, and 25–29 years) according to gender. After 8 April 1986, an increase in the number of suicide cases was observed for four days among 10–14-year-old females [15]. Using a multiplicative model of annual and monthly expected suicides, Fukutomi *et al*. determined the months in which the observed suicide rate was significantly greater than the expected rate. However, the effect of suicide reports in the media was not included in this study [13]. In summary, these studies suggested that media reports influence suicide.

Two subsequent studies concluded that the media does have an effect on suicide [5,14]. These studies used time series data and economic models, such as the Yule-Walker and Granger causality models. In particular, the Granger causality model was able to evaluate the causal relationship between suicide reports and suicide [14]. However, these studies were limited by several methodological problems. In the study by Stack, articles concerning suicide from the *New York Times *and the *Japan Times *were used as a measure of media coverage [5], even though these two newspapers have a very limited circulation in Japan. In the study by Ishii, the length of the articles on suicide in the *Mainichi *and *Asahi *newspapers was used as a measure of suicide reporting; however, suicide articles from the *Yomiuri *newspaper, which has the largest circulation in Japan, were not used [14].

Although two studies demonstrated that the media does affect suicide, we can safely say that these findings were limited and inconclusive. The study periods analyzed in these eight studies ranged from 1954 to 1986 [5,11-17]. No study has used more recent data to analyze the effect of mass media on suicide. However, several major factors that could potentially influence suicide have arisen in Japan since 1986. These include the publication of a book entitled "Suicide Manual" [18], increased Internet use, and the proliferation of web sites about suicide [19]. "Suicide Manual" was published on 4 July 1993, and shocked Japanese society [16]. As of 1 August 2005, there were more than 17,000 Japanese web sites that offered information on suicide and its methods [19].

On 11 February 2003, a 17-year-old high school senior committed suicide by carbon monoxide poisoning using a briquette. Since 2003, there have been frequent reports of young people meeting together for the first time via the Internet and committing group suicide. Carbon monoxide poisoning using a briquette has often been used as a method of group suicide, and information on this method seems to have been acquired via online suicide sites. Access to suicide information has become easier in Japan due to increased Internet availability since the 1990s. Therefore, we used recent data from 1987 to 2005 to examine whether newspaper articles about suicide and Internet use are related to the incidence of suicide in Japan.

## Methods

### Study period

The study period was from January 1987 to March 2005 (218 months).

### Data collection

Male and female monthly suicide statistics for 1987–2005 were obtained from the Vital and Health Statistics summary published by the Statistics and Information Department of the Japanese Ministry of Health, Labor, and Welfare.

Five of the most widely circulated Japanese newspapers were examined (2004 circulation figures in parentheses): *Yomiuri *(10,120,000), *Asahi *(8,250,000), *Mainichi *(3,940,000), *Nikkei *(3,020,000), and *Sankei *(2,140,000) [20]. The Nikkei Telecom 21 database covers newspaper articles from 1980 to the present. This database includes articles from the *Nikkei *and *Asahi *from as early as 1980 and articles from the *Yomiuri*,*Mainichi*, and *Sankei *from as early as September 1986, January 1987, and September 1992, respectively [21]. The *Sankei *entered the database much later and has the smallest circulation among the five papers; therefore, the *Sankei *was excluded from the analysis. Data from January 1987 to March 2005 were analyzed for the remaining four papers (*Nikkei*, *Asahi*, *Yomiuri*, and *Mainichi*). We counted the monthly number of articles with headlines that contained the keyword "suicide". The effect of newspaper articles is assumed to depend on the type of newspaper as well as on circulation. However, these four major Japanese newspapers are very similar in terms of content and reader profile; thus, only circulation was used as a factor to assess the overall impact of suicide news [14]. Specifically, the total amount of suicide news was calculated as follows: (number of articles in *Yomiuri*) × (circulation of *Yomiuri*) + (number of articles in *Asahi*) × (circulation of *Asahi*) + (number of articles in *Mainichi*) × (circulation of *Mainichi*) + (number of articles in *Nikkei*) × (circulation of *Nikkei*).

Internet use on a commercial basis began in December 1992, and the Japanese Ministry of Internal Affairs and Communications has conducted a survey on the prevalence of Internet use among households since 1996 [22]. Using these data, we calculated the monthly household Internet use in Japan between 1987 and 2005.

### Data analysis

A linear model was fitted to the data with the number of suicides in a month as a dependent variable. The independent variables were the number of newspaper articles reporting suicide in the previous month, the prevalence of household Internet use in the previous month, the national jobless rate in the previous month, and dummy variables for 12 months. To eliminate problems with multicollinearity, outliers (or influential observations), and autocorrelation, the model was fitted using the ordinary least squares (OLS) method. Variance inflation factors (VIFs) and Cook's *D-*statistics showed that there were no problems with multicollinearity or outliers (or influential observations) in either male or female subjects. However, the Darbin-Watson *d-*statistics were 0.53 in males and 0.54 in females, and the first-order autocorrelation coefficients were 0.71 in males and 0.72 in females, indicating that the OLS estimation was highly problematic [23]. To purge the data of the effects of autocorrelation, alternative autocorrelation correction methods were considered. These were the Yule-Walker (YW) estimation, iterated YW, unconditional least squares (ULS), and unconditional maximum likelihood (ML). According to Spitzer's Monte Carlo study, the YW method did as well, or better, in estimating the structural parameter when the autoregressive parameter was not too large. However, when the autoregressive parameter was large, Spitzer recommended using ML methods [24]. In view of the autoregressive parameters of our data (i.e., autocorrelation coefficients of 0.71 in male subjects and 0.72 in females), ML methods were used to fit linear models. The analysis of a time series requires that the series is stationary and that the variance or volatility of the series is constant over time. Thus, dependent variables, rather than raw data, were log transformed, and the analysis was performed [25]. As only the dependent variable was log transformed, the coefficient value shows a change in rate when there is a change of 1 unit in an independent variable. The analysis was performed using the SAS/ETS AUTOREG procedure [26].

## Results

**Figure 1:**
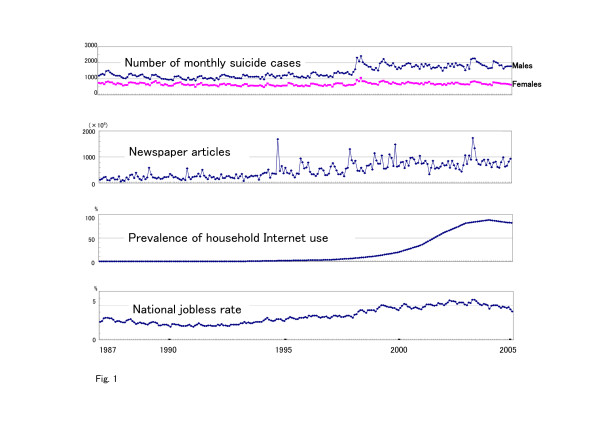
Monthly change in study variables between January 1987 and March 2005. Newspaper articles, prevalence of household internet use, and national jobless rate are the values in the previous month.

The study variables are presented in Table 1. Changes in the study variables during the study period are also shown in Fig. 1. During the study period (January 1987-March 2005), the monthly number of suicides increased by approximately 200% for males (888 to 2434) and 100% for females (479 to 1062). The mean monthly number of suicides was 1415.71 (± 352.38) for males and 672.74 (± 90.88) for females; the number of suicides among male subjects was more than double that of females. The number of newspaper reports about suicide is expressed in units of 10^6^. During the study period, the number of articles increased 30-fold from 56.9 to 1685.23 (× 10^6^).

**Table 1:** Study variables in Japan, 1987–2005

**Variables**	**Mean ± sd**	**Range**
Number of monthly suicide cases		
Males	1415.71 ± 352.38	888–2423
Females	672.74 ± 90.88	479–1062
Number of monthly suicide cases in the previous month		
Males	1415.71 ± 352.38	888–2423
Females	672.74 ± 90.88	479–1062
Newspaper articles about suicide		
in the previous month (× 10^6^)	480.33 ± 300.3	56.9–1685.23
Prevalence of household Internet use in the previous month (%)	17.65 ± 29.12	0–88.1
National jobless rate in the previous month (%)	3.43 ± 1.14	1.90–5.80

Since the launch of commercial Internet services in December 1992, the prevalence of household Internet use has increased rapidly, from 0% in December 1992 to 88.1% in March 2005. During the study period, the mean national jobless rate was 3.43% (± 1.14).

Table 2 shows the results of the unconditional ML corrections of autocorrelation according to gender. To test whether the coefficients were biased due to unequal variance in the error term between values of the independent variables (heteroscedasticity), the *Q*-statistics test for changes in variance across time was performed [26]. This test showed no possibility of heteroscedasticity in the equations for male or female subjects. With respect to males, newspaper articles on suicide (*p *< 0.001), the prevalence of Internet use (*p *< 0.05), and the national jobless rate in the previous month (*p *< 0.0001) were significant predictors of suicide (*P *< 0.001, 0.5, and 0.0001, respectively). This suggests that, after controlling for other factors, an increase in newspaper articles by 1 unit (10^6 ^distributed articles), an increase in household internet use by 1%, or an increase in the national jobless rate in the previous month by 1% will lead to increases of 0.01%, 0.16%, and 12.7%, respectively, in male suicides. The months from February to June, October and December, had significantly more suicides, respectively, than the referent month, January. Because a dummy variable was introduced for 12 months, the regression coefficient in this case refers to the difference between the mean change rate in a dependent variable in subgroups with or without the attribute shown by the dummy variable [27]. Thus, with regard to March, April, May, June, and October, the *β*-values were 0.1127 (*P *< 0.0001), 0.1169 (*P *< 0.0001), 0.1461 (*P *< 0.0001), 0.0853 (*P *< 0.000), and 0.0484 (*P *< 0.01), suggesting that, after controlling for other variables, March, April, May, June and October showed 11.27%, 11.69%, 14.61%, 8.53%, and 4.84% increases in suicide incidence, respectively, compared to January. With regard to February and December, the *β*-values were -0.0712 (*P *< 0.0001) and -0.0480 (*P *< 0.01), suggesting that, after controlling for other variables, February and December showed 7.12% and 4.80% decreases in the incidence of suicide, respectively, compared to January.

**Table 2:** Effects of publicized suicide stories and Internet use on the monthly suicide rate, 1987–2005 (*n *= 218)

	**Males**			**Females**		
	Coefficient	Standard Error	*t-*statistics	Coefficient	Standard Error	*t-*statistics

Intercept	6.6852****	0.0650	102.82	6.3286****	0.0744	85.10
Newspaper suicide reports in the previous month	0.0001***	0.0000	3.44	0.0001***	0.0000	3.97
Prevalence of Internet use in the previous month	0.0016*	0.0008	1.98	0.0001	0.0009	0.09
National jobless rate in the previous month	0.1270****	0.0208	6.09	0. 0172	0.0238	0.72
February	-0.0712****	0.0149	-4.78	-0.0722****	0.0165	-4.38
March	0.1127****	0.0174	6.49	0.1434****	0.0193	7.44
April	0.1169****	0.0188	6.21	0.1703****	0.0209	8.16
May	0.1461****	0.0196	7.45	0.1975****	0.0217	9.09
June	0.0853***	0.0202	4. 22	0.1211****	0.0224	5.41
July	0.0715	0.0206	3.47	0.1068****	0.0228	4.68
August	0.0159	0.0201	0.79	0.0516*	0.0223	2.31
September	0.0207	0.0195	1.06	0.0243	0.0216	1.13
October	0.0484**	0.0184	2.62	0.0518*	0.0204	2.53
November	-0.0056	0.0167	-0.33	0.0245	0.0185	1.32
December	-0.0480**	0.0163	-2.95	-0.0117	0.0181	-0.64
	*R*^2 ^= 0.96, DW = 1.81			*R*^2 ^= 0.80, DW = 2.02		

*P < 0.05, **P < 0.01, ***P < 0.001, ****P < 0.0001

With respect to female subjects, the number of newspaper articles about suicide in the previous month was a significant predictor of suicide (*P *< 0.001). These results suggest that, after controlling for other factors, an increase in the number of newspaper articles by 1 unit (× 10^6 ^distributed articles) will lead to an increase of 0.01% in the incidence of suicide among females. The months from March to August, and October, had significantly more suicides, respectively, than the referent month, January. In March, April, May, June, July, August, and October, the *β*-values were 0.1434 (*P *< 0.0001), 0.1703 (*P *< 0.0001), 0.1975 (*P *< 0.0001), 0.1211 (*P *< 0.0001), 0.1068 (*P *< 0.0001), 0.0516 (*P *< 0.05) and 0.0518 (*P *< 0.05), suggesting that after controlling for other variables, these months showed 14.34%, 17.03%, 19.75%, 12.11%, 10.68%, 5.16% and 5.18% increases in suicide cases, respectively, compared to January. With regard to February, the *β*-value was -0.0722 (*P *< 0.0001), suggesting that after controlling for other variables, February showed a 7.22% decrease in suicide incidence compared to January.

To determine whether the association between suicides and media reports (previous month) for males and females (Table 2) was a spurious relationship between suicide deaths in one month and suicide deaths in the previous month (autocorrelation), we preformed additional multiple regression analyses. We used (1) the same model as in Table 2 except the media reports were from the same month instead of the previous month and (2) a model including suicides in the previous month as an independent variable in addition to those in Table 2. In the first analysis, the number of newspaper articles on suicide (same month) was a significant predictor of suicide among males (B = 0.0001, *t *= 3.46, *p *< 0.0001) and females (B = 0.0001, *t *= 4.05, *p *< 0.0001). In the second analysis, the number of suicides (previous month) was a significant predictor of suicide among males (B = 0.0001, *t *= 4.29, *p *< 0.0001) and females (B = 0.0001, *t *= 3.49, *p *= 0.0006). Furthermore, the other significant variables were exactly the same as those shown in Table 2 [*i.e*., prevalence of internet use (previous month), national jobless rate (previous month), month dummies for males, and month dummies for females]. Thus, there should be no problems related to autocorrelation in Table 2.

## Discussion

Based on data from 1987 to 2005 in Japan, we evaluated the effect of newspaper articles about suicide and Internet use on the occurrence of suicide, according to gender. Several conclusions can be drawn from our findings. Because the *β*-values for newspaper articles about suicide were positive and significant in males (*β *= 0.0001, *P *< 0.001) and females (*β *= 0.0001, *P *< 0.001), it is likely that newspapers articles affect the occurrence of suicide in both genders. These results are consistent with two previous studies based on time series data [5,14]. With respect to the association between Internet use and suicide, our results differed for males and females. The *β*-values for the prevalence of Internet use were positive and significant for males (*β *= 0.0016, *P *< 0.05), but not for females (Table 2). These results may be the result of gender-based differences in Internet use. According to the Japan Policy Agency, the numbers of suicide cases and victims among people meeting for the first time via the Internet were, respectively, 19 and 55 in 2004 and 34 and 91 in 2005, showing an upward trend [28]. A survey on Internet use in the city of Yamato, which is near the Tokyo metropolitan area, revealed several differences between males and females with respect to their attitudes toward the Internet and Internet use [29]. At the time of the study in 2000, 47.52% of males had access to the Internet compared to only 36.1% of females. Furthermore, female respondents were less willing to express their opinions on the Internet, and indicated that they found computers difficult to use. According to a study in 2003 conducted by the National Institute of Communications Technology (NICT), Internet use was higher among 12–74-year-old males (55.7%) than females (49.9%) [30]. These findings suggest that Internet use and newspaper articles on suicide have a differential effect on suicide incidence in males and females.

This is the first study to examine the association between Internet use and the number of suicides in Japan. The NICT survey reported that the mean time spent online was longer than the mean time spent reading newspapers in 2004 (37 versus 31 min per day, respectively) [31]. The time spent online is expected to increase in Japan until Internet use becomes as saturated as it is in the United States, meaning that the Internet will continue to be a potential predictor of the suicide rate in Japan [31].

The *β*-coefficient for the national jobless rate was only positive and significant in males (*β *= 0.1270, *P *< 0.0001; Table 2). Several previous studies have reported the association between economic strain and suicide [14,32]. Our results show that the unemployment rate was a significant predictor of suicide among males, but not females. Inoue *et al*. recently reported that the annual suicide rate among Japanese males was significantly correlated with the annual unemployment rate, whereas the suicide rate for Japanese females was not associated with unemployment [33]. Although our results are consistent with these previous findings, further investigation is required to determine why unemployment is not a predictor of suicide in females.

Certain months of the year were also significant predictors of suicide (Table 2). The β-coefficients for the months of March to June were significant and positive for both males and females, and July was also significant and positive for females (Table 2). Based on combined data for males and females, Stack reported that the months from March to June were positive predictors of suicide in Japan [5]. Similarly, Fukutomi *et al*. reported that the number of suicides was high in April and low in December [13]. Our findings are consistent with these previous studies.

We attempted to improve upon the methodological issues and limitations of previous studies. First, of the eight studies investigating the effect of media reports on suicide in Japan [5,11-17], only two analyzed the time series data using the appropriate models [5,14]. To produce a more reliable assessment of this issue, we analyzed time series data using a linear model. We also used suicide reports and the national jobless rate during the previous month to establish a temporal relationship between stimuli (suicide news) and response (suicide). With respect to suicide data, Phillip and Bollen pointed out the importance of daily data. Given that those who are influenced by news media will commit suicidal acts on a daily basis, it might be difficult to accurately evaluate stimuli (suicide news) and response (suicide) relationships on the basis of monthly data. Thus, Bollen and Phillips used daily data instead of monthly data in their analyses [34,35]. However, we were required to rely on monthly data because daily data on newspaper articles related to suicide and Internet use were not available. Furthermore, the model used in our study does not consider other possible or known risk factors for suicide, such as the rise in alcohol use, drug use, domestic violence, and the poor provision of mental health care. More sophisticated models based upon daily data including these variables need to be tested in future studies.

Previous studies used articles from English language newspapers circulated in Japan or from a limited number of the larger domestic Japanese papers [5,14]. To improve the accuracy of our measure, we used articles on suicide from the four largest domestic newspapers. However, articles were selected based on the keyword "suicide" in the headline. This approach may have excluded articles that discussed suicide but were not specifically suicide reports, such as editorials, commentaries, and fictional stories. Likewise, this approach may have included non-relevant articles that used "suicide" in another context. In addition, it is possible that some relevant articles were excluded because the headlines may have cited the method of suicide rather than the keyword "suicide". In this connection, we need to refer to news articles related to "9–11" in 2001. Owing to the worldwide reporting of suicide bombing attacks in recent years, one might think that this approach has led to biased results. However, suicide and suicide bombing attacks are pronounced and expressed quite differently in Japanese. Thus, in reality, there was no overlap between articles on "suicide" versus "suicide bombing (attacks)". Finally, other potentially important factors, such as front-page coverage or photos or illustrations of the deceased, were not considered when searching for newspaper articles about suicide. Future studies will require a more refined measure of the impact of newspaper articles.

Detailed information on Internet use has been available since 2001 [30], allowing us to include the prevalence of household Internet use in our study. However, the extent of exposure to Internet news was not determined, and thus our results concerning Internet use should be interpreted cautiously. The Internet may be used for leisure or communication purposes rather than reading the news. Perhaps only a small percentage of Internet users were exposed to suicide reports, and an even smaller proportion of these were influenced by the news. In addition, help organizations now offer help via the Internet and, thus, Internet use may have had a preventive impact on the number of suicides that was not evaluated in this study. Although worldwide Internet use has increased in the observed time period, suicide incidence has decreased in many countries. Therefore, it is important to determine if Japanese web sites devoted to suicide or suicide prevention differ from web sites in other countries, and whether a decrease in suicide incidence is accompanied by an increase in Internet use. Further studies will be required to thoroughly evaluate the positive and negative effects of online suicide reports.

We examined whether suicide incidence increased more rapidly before or after 1992 and the advent of the Internet; however, no difference in the increase of suicide incidence was observed. Future studies will require a more precise measure of exposure to suicide reports and related topics via the Internet.

It can be expected that the Internet and newspaper articles have different effects on people, according to age and gender. An article from *Internet Watch *reported that the time spent online per month increased by 58% in the 2- 12-year-old age group, 44% in the 50- 59-year-old age group, and 3% in the 16- 19-year-old age group between March 2001 and 2002 [31]. Although the mean time spent online has increased more than that spent reading newspapers in 2004, people in their 60s or older spend more time reading newspapers than going online [30]. Our study design was not able to evaluate the differential influence of news articles and Internet use on suicide incidence in terms of gender and age. This is an area for future study.

## Conclusion

We examined the relationship between newspaper articles about suicide and Internet usage and the number of suicides in Japan from 1987 to 2005. We found the following: (1) newspaper articles about suicide are a predictor of suicide for both male and female subjects, which is consistent with previous findings, and (2) Internet use was a predictor of suicide among males, who spent longer hours online, as compared to females. More research is required to verify our findings regarding Internet use and suicide incidence.

## Competing interests

The author(s) declare that they have no competing interests.

## Authors' contributions

AH has made substantial contributions to conception and design, analyzed and interpreted data and drafted the manuscript. KT has been involved in drafting the manuscript or revising it critically for important intellectual content and has given final approval of the version to be published. TA has been involved in collecting and entering data. All authors read and approved the final manuscript.

## Pre-publication history

The pre-publication history for this paper can be accessed here:


